# Changes in cardiovascular health following ischemic preconditioning in older adults with knee osteoarthritis

**DOI:** 10.14814/phy2.70675

**Published:** 2025-12-08

**Authors:** Joseph Lupa, Shraddha Sudhir, Nikou Nikoumanesh, Lindsay S. Hannigan

**Affiliations:** ^1^ Department of Physical Therapy University of Illinois at Chicago Chicago Illinois USA

**Keywords:** blood pressure, cardiovascular health, ischemic preconditioning, knee osteoarthritis, older adults

## Abstract

Ischemic preconditioning (IC) has been studied for its cardiovascular benefits, yet little is known about its effects in older adults with knee osteoarthritis (KOA). The purpose of this study is to explore the impact of a single IC session on markers of cardiovascular health and metabolic efficiency in adults with KOA. Participants were assigned using block randomization to either an IC group or a sham control group. Blood pressure (BP), augmentation index (AIx), pulse wave velocity (PWV), and walking economy (WE) measurements were taken. Both groups were subjected to five cycles of occlusion followed by reperfusion for 5 min each, for a total of 50 min. For the IC group the cuff was inflated to 225 mmHg to fully occlude arterial blood flow while the sham group received cuff inflation at 25 mmHg that did not induce ischemia. BP, AIx, PWV, and WE were measured after the intervention. A small but significant increase in systolic and diastolic blood pressure was observed in the IC group compared to the control group. However, no significant changes were found in PWV, AIx, and WE. These findings suggest that even a single session of IC may influence blood pressure regulation in older adults with KOA, though its effects on vascular function remain unclear. This study provides insight into the effects of IC on blood pressure in older adults with KOA. Further studies are needed to explore the underlying mechanisms and potential therapeutic applications of repeated sessions of IC in older individuals with joint pathology.

## 1 INTRODUCTION

Knee osteoarthritis (KOA) is a degenerative disease of the knee joint that leads to increased disability. KOA is particularly prominent in older adults with over 80% of adults older than 65 years showing radiographic evidence of KOA (Geng et al., 2023; Lim & Al‐Dadah, 2022; Park et al., 2023; Sharma, 2021; Silverwood et al., 2015). KOA is increasingly recognized as a systemic process which generally develops over years with its progression influenced by comorbidities, such as cardiovascular disease (CVD), obesity, pain perception, and other psychological stress (Roos & Arden, 2016). Older adults with KOA experience higher rates of cardiovascular comorbidities than the general population, often driven by overlapping risk factors such as physical inactivity (Swain et al., 2020).

Walking exercise is a widely recommended conservative intervention to help reduce the progression of KOA and relieve symptoms (Charlesworth et al., 2019; Kolasinski et al., 2020). Contrary to misconceptions that exercise leads to KOA, physical activity is more protective than detrimental for the knee joint (Alentorn‐Geli et al., 2017; Felson et al., 2007; Mühlbauer et al., 2000; Raposo et al., 2021). Despite the benefits of exercise, most individuals do not participate in exercise without clinician oversight (Nicolson et al., 2018; Pisters et al., 2010). Lack of physical activity and exercise may also be exacerbated by pain and limited mobility from KOA, which can lead to worsening cardiovascular health (Swain et al., 2020). This creates a vicious cycle of negative health outcomes with worsening KOA promoting worsening CVD (Park et al., 2023). There is a need to explore interventions other than physical activity to improve function and health in individuals with KOA.

**FIGURE 1:**
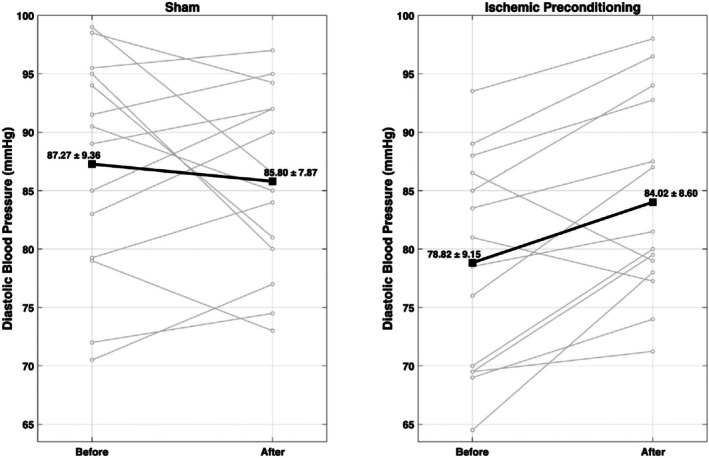
Participants in the ischemic preconditioning (IC) group demonstrated increased diastolic blood pressure following IC (+5.2 mmHg) while those in the sham group demonstrated no significant change (−1.8 mmHg).

**FIGURE 2:**
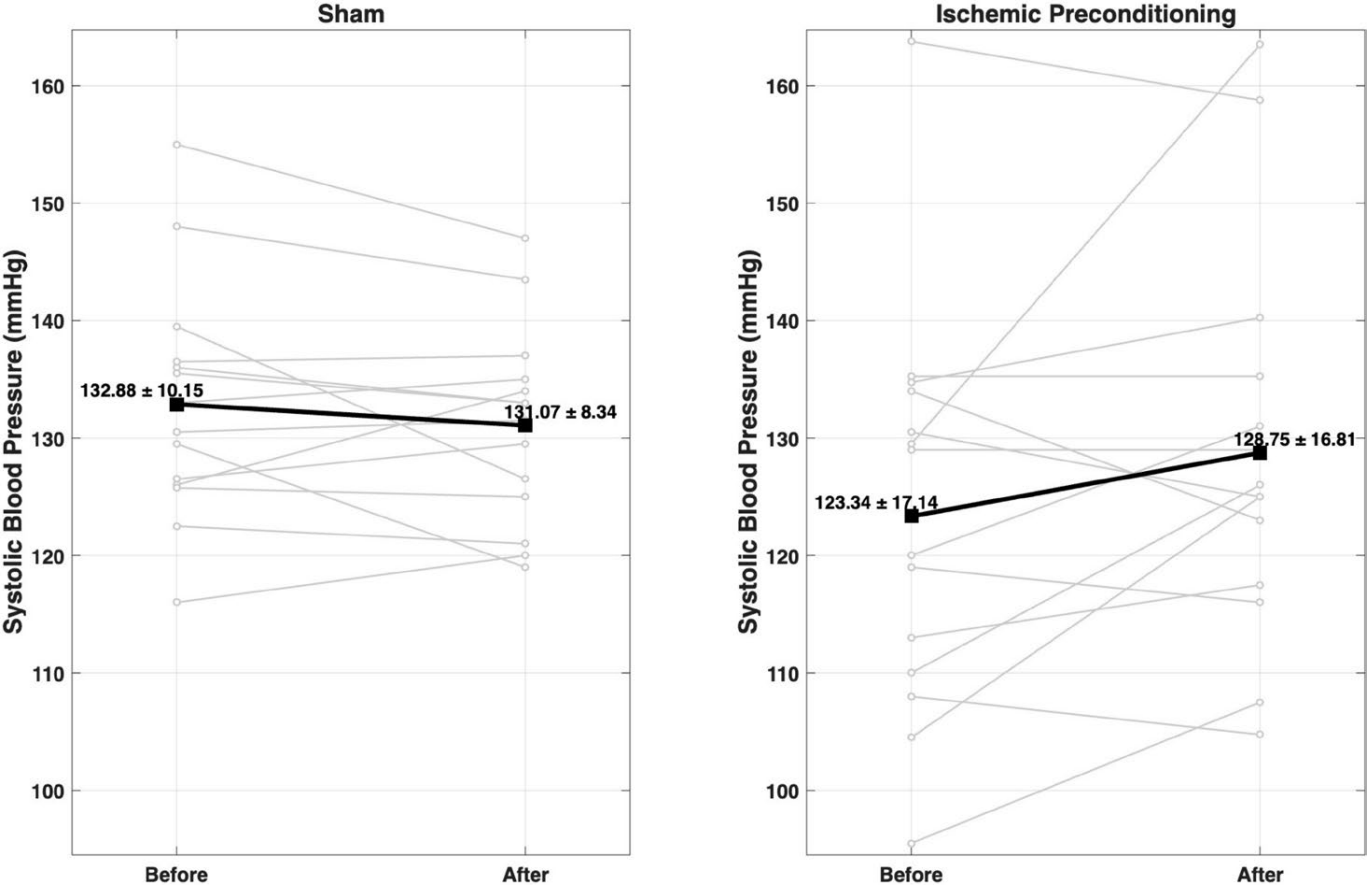
Participants in the ischemic preconditioning (IC) group demonstrated increased systolic blood pressure following IC (+5.4 mmHg) while those in the sham group demonstrated no significant change (−1.8 mmHg).

Ischemic preconditioning (IC) is a noninvasive intervention that involves intermittent restriction and restoration of blood flow to the limb via an inflatable cuff. A typical IC protocol involves repetitive cycles of ischemia (cuff occlusion) and reperfusion (cuff deflation) while resting supine (Incognito et al., 2016; Marocolo et al., 2023; Salvador et al., 2016). IC increases exercise performance (Cheng et al., 2021; Cruz et al., 2015; Salagas et al., 2022), induces changes which alter flow mediated dilation in the treated and contralateral limb (Hyngstrom et al., 2018; Jones et al., 2014), increases muscle activation (Hyngstrom et al., 2018; Sudhir et al., 2024), and protects cardiac tissue from more prolonged bouts of ischemia (Eisen, 2004). Repeated IC exposure has been associated with improvements in vascular function in individuals with chronic stroke (Hyngstrom et al., 2020), suggesting that the intervention may modulate cardiovascular responses even in clinical populations with impaired vascular health. These findings provide a rationale to examine whether a single IC session may also influence cardiovascular measures in older adults with KOA, a group who also has elevated cardiovascular risk. IC has demonstrated potential benefits across various clinical populations and is safe, affordable, and accessible in both clinical and home settings (Figures 1 and 2).

There is limited information regarding the effects of IC in individuals with KOA, particularly in relation to both cardiovascular function and walking economy. Walking economy, a measure of the energy cost of ambulation, is influenced by multiple factors including muscle efficiency, joint mechanics, and hemodynamic regulation. Impairments in vascular health may contribute to reduced walking efficiency in older adults with KOA through altered oxygen delivery or peripheral circulation. Because IC transiently alters blood flow and vascular tone, it is reasonable to explore whether these acute cardiovascular changes may also be accompanied by alterations in walking economy.

Therefore, this study aimed to examine whether a single session of IC could acutely influence cardiovascular markers and walking economy in adults with KOA. To characterize cardiovascular function, we assessed central blood pressure, pulse wave velocity, and augmentation index. We hypothesized that a single session of IC would improve cardiovascular markers, including blood pressure and arterial stiffness, and lead to improved walking economy when compared to sham in older adults with KOA.

## 2 METHODS

Twenty‐eight adults aged 50 years and older (50–81 years) with KOA, as defined by the American College of Rheumatology (Altman et al., 1986) in at least one limb, volunteered for this study (Table 1). Individuals with a history of knee replacements, corticosteroid shots within the last 6 months, and/or neuromuscular disorders which affected their gait were excluded from the study. Participants were block randomized to either IC (225 mmHg) or a sham treatment (25 mmHg) with participants from each group being matched for age and sex. All participants provided written informed consent which was approved by the university's institutional review board. The study was conducted in accordance with the Declaration of Helsinki and approved by the Institutional Review Board of the University of Illinois at Chicago (2024–0021). Participant demographics are reported in Table 1.

**TABLE 1:** Participant average and standard deviation demographics in the IC and sham intervention groups.

Variable	IC	Sham	*p*‐value
*N*	14 (9 M/5F)	14 (9M/5F)	
Age (years)	63.1 (8.9)	65.3 (9.8)	*p* = 0.53
Height (cm)	172.5 (13.8)	173.5 (9.9)	*p* = 0.75
Mass (kg)	90.3 (17.2)	93.2 (27.2)	*p* = 0.85
BMI (kg/m^2^)	30.6 (7.0)	30.5 (6.9)	*p* = 0.975

Abbreviations: cm, centimeters; kg, kilograms; m, meters.

Participants arrived at the lab and rested supine for 30 min following providing written informed consent. After the rest period, resting heart rate, central blood pressure, augmentation index (AIx), and carotid to femoral pulse wave velocity (PWV) were measured using a SphygmoCor XCEL system and tonometer (SphygmoCor; AtCor Medical, West Ryde, New South Wales, Australia). Participants sat upright and a brachial cuff was placed on the participants' right side to measure central blood pressure. AIx was calculated by taking the ratio of aortic pulse pressure to augmented aortic pressure and expressed as a percentage (Atcor, 2016). To measure PWV, participants were positioned supine on a therapy table and an inflatable cuff was placed around the right thigh to measure femoral pulse, and the location of the participants' strongest carotid pulse was palpated and marked. Distances between the carotid pulse and sternal notch, sternal notch to the superior border of the cuff, and between the sternal notch and the location of the strongest femoral pulse, were measured. Measurements were taken over 10 s which led to approximately 10 wave forms per measurement, which were checked for quality and averaged to give a single measurement by the SphygmoCor XCEL System (Atcor, 2016). In cases where the femoral pulse could not be strongly felt, its location was estimated (Atcor, 2016). All measures were recorded by the same researcher in triplicate and the average of the two nearest PWV recordings was used.

Participants then walked on a dual‐belt instrumented treadmill (Bertec, Columbus, Ohio, USA) at a self‐selected walking speed for 6 min (6MWT) During the 6MWT, participants were fitted with a portable breath‐by‐breath open circuit indirect calorimetry system (Caliber Biometrics). The treadmill speed was gradually increased until the participant confirmed that the pace felt challenging yet safe to maintain, at which point the treadmill's speed was set for the duration of the 6MWT. Gait speed and total oxygen consumption from indirect calorimetry were recorded. Walking economy (WE) was calculated by total oxygen consumed during the 6MWT normalized to body mass, and divided by distance covered during the 6MWT (mL/kg/m) (Huang et al., 2021).

Following baseline measures, participants rested supine for the intervention and were fitted with a rapid inflation cuff on the proximal thigh of the involved knee for IC or sham protocols. The IC protocol lasted for 50 min, which included 5 cycles of 5 min of full occlusion (225 mmHg) followed by 5 min of reperfusion. The sham protocol followed the exact same procedures; however the cuff was inflated to 25 mmHg during occlusion periods so that the participants would feel the pressure without occluding blood flow. These protocols follow those that have been previously published (Hyngstrom et al., 2018; Sudhir et al., 2024). Immediately following the IC/sham intervention, all baseline measures were repeated in the same order.

### 2.1 Statistical analysis

Our study was powered to identify a 0.5 m/s decrease in PWV, corresponding to a standardized effect size of 1.02 based on published data (Zagidullin et al., 2016). Therefore, we required 14 individuals per group, for a total of 28 participants to detect the effect size of 0.8 with a 20% attrition rate for 80% power with a Type I error at 5% using a two‐sided paired *t*‐test. From a sample size of 28, we can obtain an effect size of 1.02 for independent t‐tests when change scores are compared between sham and IC.

All dependent variables were assessed for normality using a Shapiro‐Wilk test. Change scores were calculated for all variables by subtracting the post‐treatment measures from the pre‐treatment measures. Body mass index (BMI) was calculated for all participants (kg/m^2^). Pearson's correlation coefficients were computed to assess associations between BMI and change scores for SBP, DBP, PWV, and AIx. A multiple analysis of variance was used to compare changes in dependent variables based on group (IC/sham). All analyses were performed using SPSS (version 28, IBM, Armonk, NY) with an alpha level of 0.05 set a priori. Mean differences were calculated for all pre‐post changes.

## 3 RESULTS

All variables were normally distributed (*p* > 0.05). There was a statistically significant increase in systolic blood pressure (mean difference = 5.4 mmHg) and diastolic blood pressure (mean difference = 5.2 mmHg) in the IC group from pre‐treatment to post‐treatment (Table 2). There were no significant changes in resting heart rate, PWV, AIx, or walking economy in the IC group. No significant correlations were found between BMI and changes in any cardiovascular measures (all *p* > 0.05). There were no significant changes in any variables in the sham group.

**TABLE 2:** Average and standard deviations for all dependent variables for both intervention groups before and after intervention with mean differences (MD).

	IC			SHAM		
	Pre	Post	MD	Pre	Post	MD
Systolic pressure (mmHg)	123 (17)	129 (17)	5.4 (12)^a^	133 (10)	131 (8.3)	−1.8 (5.8)
Diastolic pressure (mmHg)	78.8 (9.2)	84.0 (8.6)	5.2 (5.7)^a^	87.3 (9.4)	85.8 (7.9)	−1.5 (7.8)
Heart rate (bpm)	64.6 (7.3)	60.9 (9.6)	−3.8 (6.5)	67.4 (8.7)	65.1 (7.0)	−2.3 (5.7)
Augmentation index (%)	32.6 (10)	29.0 (10.6)	−3.6 (7.7)	31.9 (12)	30.1 (7.5)	−1.8 (11)
Pulse wave velocity (m/s)	8.47 (1.5)	8.68 (2.8)	0.21 (0.49)	9.38 (1.4)	9.23 (1.2)	−0.14 (1.2)
Walking economy (mL/kg/m)	0.331 (0.09)	0.302 (0.14)	−0.02 (0.06)	0.312 (0.08)	0.291 (0.06)	−0.02 (0.05)

Abbreviations: bpm, beats per minute; IC, ischemic preconditioning; MD, mean difference.

^a^ Indicates significant differences when compared to sham (SBP‐*p*=0.028, DBP‐*p*=0.008).

## 4 DISCUSSION

The purpose of this study was to explore if a single IC intervention influenced cardiovascular function in adults with KOA. The results suggest a small hypertensive effect after a single session of IC. This is contrary to several studies which have shown that younger athletes demonstrate a hypotensive response to IC (Foster et al., 2011; Panza et al., 2019). Adults with KOA in our study demonstrated an acute increase in blood pressure comparable to the hemodynamic response during exercise. Emerging evidence indicates that repeated IC sessions may replicate some of the long‐term cardiovascular benefits of regular exercise (Zhao et al., 2018). IC and exercise activate overlapping physiological mechanisms, including increases in heat shock protein expression (Zhao et al., 2018), attenuation of oxidative stress (Morihira et al., 2006), and the induction of cytoprotective and anti‐inflammatory signaling pathways (Konstantinov et al., 2004; Liu et al., 2016; Zhao et al., 2018). Taken together, these parallels offer a mechanistic framework through which the acute hemodynamic response we observed may reflect an early‐stage engagement of the shared adaptive processes of exercise and IC.

It is also possible that the hypertensive response observed after IC in our cohort may be related to acute sympathetic activation and temporary increases in peripheral vascular resistance that accompany ischemia–reperfusion. While younger adults often demonstrate post‐conditioning hypotension (Tong et al., 2019), older adults typically exhibit reduced arterial compliance and diminished baroreflex sensitivity (Monahan, 2007), which may attenuate the compensatory vasodilatory response and result in a short‐term elevation in blood pressure. This could explain our results and further highlights the need to study the effects of repeated sessions of IC.

Although we did not observe improvements in walking economy, including this outcome allowed us to assess whether short‐term cardiovascular changes following IC might relate to whole‐body metabolic efficiency. The absence of change suggests that acute hemodynamic responses may not translate into immediate alterations in walking economy, though this relationship may warrant further study under repeated or longer‐term IC protocols as repeated sessions of IC have been shown to improve brachial artery flow mediated dilation in stroke survivors, which suggests that repeated sessions of IC may improve underlying endothelial function generally (Hyngstrom et al., 2020). The proposed mechanism of endothelial protection is an increase in endothelial nitrate oxide synthase activity which has been shown in both exercise and IC (Hambrecht et al., 2003; Hyngstrom et al., 2020; Liang et al., 2015). Taken as a whole this suggests that when administered over an extended period, repeated IC may contribute to reductions in baseline blood pressure, potentially through shared physiological pathways such as improved endothelial function and autonomic regulation (Zhao et al., 2018). Our findings suggest that, while a single session of IC is insufficient to induce sustained changes in baseline cardiovascular markers, it may represent the initial activation of physiological pathways that—when repeatedly stimulated—could lead to meaningful cardiovascular adaptations in those with KOA. Further research should continue investigating the effect of IC on cardiovascular function in those with symptomatic KOA.

Although there were changes in blood pressure, there were no changes in any other cardiovascular marker after a single session of IC. Acute IC exposure may not be sufficient to alter arterial stiffness, resting heart rate, or walking economy in older adults with KOA, similar to a lack of change shown in young adults (Müller et al., 2019). IC may be an effective strategy for reducing arterial stiffness, as assessed by PWV, in individuals with elevated blood pressure when applied across multiple sessions (Ghimire et al., 2025). In addition, a single IC session can increase muscle power in older adults with KOA and stroke survivors (Hyngstrom et al., 2018; Sudhir et al., 2024), however these neuromuscular improvements may not translate into immediate gains in walking economy (Sudhir et al., 2024).

Participants were matched by age and sex to reduce the impact of confounding variables. Nonetheless, the remote application of ischemic conditioning (IC) to the thigh may have been insufficient in either proximity or physiological intensity to induce measurable cardiovascular adaptations following a single session, particularly in older adults. It should also be noted that approximately 40% of participants reported using antihypertensive, beta‐blocker, or statin medications, which may have influenced cardiovascular responses; medication use was similar between groups and so is unlikely to account for the observed effects. Moreover, variability in disease severity may have influenced responses, as the American College of Rheumatology criteria used for inclusion do not incorporate radiographic assessment and thus do not provide a Kellgren–Lawrence (KL) grade. Additionally, because the ACR clinical criteria for symptomatic KOA do not include a minimum duration of symptoms, variability in disease duration across participants may have influenced responses to the intervention. Lastly, we did not account for differences in participants' baseline physical activity or overall fitness level, which represents a potential confounding variable

In conclusion, this study was the first to investigate changes in cardiovascular function in individuals with KOA following a single IC session. Our data support that a single session of IC can increase blood pressure but did not affect any other cardiovascular measures. Acute exposure may not be sufficient to impact cardiovascular function, and repeated exposures may be necessary. Future research should aim to elucidate the mechanisms underlying any age‐related differences and evaluate the long‐term efficacy and safety of IC as a viable strategy to promote cardiovascular health in this high‐risk population.

## FUNDING INFORMATION

American Heart Association through UICHeart: University of Illinois Undergraduate Mentoring and Experience in Heart Research (DOI: https://doi.org/10.58275/AHA.24IAUST1201236.pc.gr.189965).

## CONFLICT OF INTEREST STATEMENT

The authors declare no conflict of interest.

## Data Availability

Aggregate data will be made available based on request to the primary author.
